# Evaluation of the Bitterness-Masking Effect of Powdered Roasted Soybeans

**DOI:** 10.3390/foods5020044

**Published:** 2016-06-18

**Authors:** Yoshimasa Makita, Tomoko Ishida, Noriko Kobayashi, Mai Fujio, Kyoko Fujimoto, Rina Moritomo, Jun-ichi Fujita, Shin-ichi Fujiwara

**Affiliations:** 1 Department of Chemistry, Osaka Dental University, 8-1 Kuzuhahanazono-cho, Hirakata, Osaka 573-1121, Japan; fujiwara@cc.osaka-dent.ac.jp; 2 Osaka Dental University, 8-1 Kuzuhahanazono-cho, Hirakata, Osaka 573-1121, Japan; s11010@stu.osaka-dent.ac.jp (T.I.); s11048@stu.osaka-dent.ac.jp (N.K.); s11096@stu.osaka-dent.ac.jp (M.F.); s11098@stu.osaka-dent.ac.jp (K.F.); s11113@stu.osaka-dent.ac.jp (R.M.); 3 Department of English, Osaka Dental University, 8-1 Kuzuhahanazono-cho, Hirakata, Osaka 573-1121, Japan; fujita-j@cc.osaka-dent.ac.jp

**Keywords:** powdered roasted soybean, bitterness, masking effect, quinine hydrochloride, denatonium benzoate

## Abstract

The masking of bitterness is considered important because many pharmaceutical compounds have a bitter taste. The bitterness-masking effect of powdered roasted soybeans (PRS) was investigated using a bitter taste sensor. PRS was revealed to significantly suppress the bitterness of quinine hydrochloride and denatonium benzoate. Furthermore, the bitterness-masking mechanism of PRS extracts was evaluated using dynamic light scattering. These results showed that the extracted suspension consisted of particles that were several hundreds of nanometers in size. Analysis of the PRS extracts by nuclear magnetic resonance spectroscopy indicated that denatonium benzoate was entrapped in the PRS extracts. Thus, PRS may be useful as a bitterness-masking agent in orally administered pharmaceuticals.

## 1. Introduction

The five basic tastes comprise sweet, salty, sour, bitter, and umami. In particular, sour and bitter tastes are generally unfavorable and avoided by humans because toxic substances often taste bitter [1,2]. Bitter-tasting foods are not preferred in most cases, with a few exceptions including coffee, beer, and wine. Even though humans are averse to bitter-tasting substances, pharmaceutical compounds with physiological benefits often taste bitter. Therefore, the masking of bitterness is considered important in food processing and pharmacology.

Several bitterness-masking methods have been developed, such as the addition of other tastes and flavors to suppress bitter tastes [3,4,5]. Bitterness can also be masked by applying antagonists for bitter taste receptors (T2Rs), which are classified as G protein–coupled receptors [6,7,8,9]. Moreover, coating and encapsulating are often used in the pharmaceutical industry to mask the bitterness of drugs [10,11,12,13,14,15,16,17]. The formation of inclusion complexes between cyclodextrin and various substances can be used to mask bitterness [18,19,20]. Furthermore, phosphatidic acid and its lipoprotein derivative formed by interactions with β-lactoglobulin are reported to suppress the bitterness of quinine [21]. In addition, amino acid derivatives are low-molecular-weight bitterness-masking compounds [22]. In many cases, however, the bitterness-masking mechanisms of these compounds have not been elucidated. For food processing, these compounds must be harmless; therefore, identifying safe bitterness-masking agents originating from foods is desirable.

Powdered roasted soybeans (PRS), called kinako in Japan, are a traditional Japanese food. Each 100 g of PRS is composed of 39 g of protein, 25 g of total lipid, 30 g of carbohydrate, and small amounts of minerals and vitamins [23]. PRS is inexpensive and easily handled in its powder form. Therefore, we thought that if a bitterness-masking effect could be confirmed, PRS would be a useful bitterness-masking agent. In a previous study, we used a sensory evaluation test to investigate the bitterness-masking effect of PRS [24]. This test revealed that PRS masks many components of bitterness, and it was particularly effective in masking the bitterness of hydrophobic components, such as quinine hydrochloride (QH) and denatonium benzoate (DB). However, other powdered foods such as starch did not mask the bitterness in this test.

In this study a bitter taste sensor (Intelligent Sensor Technology, Inc., Kanagawa, Japan), a powerful tool for quantifying bitterness-masking [25,26,27,28,29,30,31], was used to confirm the bitterness-masking effect of PRS previously found in sensory tests. Furthermore, the bitterness-masking mechanism was evaluated by extracting the masking components from PRS and assessing them using dynamic light scattering (DLS) and nuclear magnetic resonance (NMR) analyses.

## 2. Materials and Methods

### 2.1. Reagents

Powdered roasted soybeans were obtained from Kawamitsu Bussan Co., Ltd. (Tokyo, Japan), quinine hydrochloride was obtained from Nacalai Tesque Inc. (Kyoto, Japan), and denatonium benzoate was obtained from Tokyo Chemical Industry Co., Ltd. (Tokyo, Japan).

### 2.2. Bitter Taste Sensing

A taste sensing system (TS-5000Z, Intelligent Sensor Technology, Inc., Kanagawa, Japan) was used to measure bitter ingredients [25,26,27]. The sensor has a working electrode with a lipid/polymer membrane for sensing and a reference electrode. Changes in the membrane potential generated when the working electrode is immersed in each sample are measured. The bitterness of 0.2 mg/mL QH solution and 0.02 mg/mL DB solution were measured by the change in the membrane electric potential when the bitter ingredients were absorbed into the membrane. To measure the reference potential (Vr), the sensor electrode was immersed in a 30 mM KCl solution. Subsequently, the sensor electrode is immersed in the sample solution to measure the membrane potential (Vs). The difference between these potentials, Vs − Vr, is defined as the sensor output. Solutions containing masking ingredients PRS (10 or 15 mg/mL) and OH (0.2 mg/mL) or DB (0.02 mg/mL) were prepared, and the sensor output was obtained for each sample.

### 2.3. Dynamic Light Scattering

PRS (10.0 g) was added into 100 mL of chloroform. The heterogeneous solution was stirred for 1 h, and then filtered. The organic layer was evaporated and 1.0 g of residue was obtained. This residue was purified using gel filtration chromatography on Sepharose 4B (GE Healthcare Bio-Science KK, Tokyo, Japan). The PRS-extracts were dissolved in phosphate buffered saline solution (pH 7.4, 0.1 mM) and evaluated by DLS. The diameters of the particles were measured using a Zetasizer Nano ZS ZEN3600 (Malvern Instruments Ltd., Worcestershire, UK).

### 2.4. Nuclear Magnetic Resonance

DB (5.0 mg), extracted PRS (10.0 mg), or DB (5.0 mg) and extracted PRS (10.0 mg) were dissolved in 0.6 mL of deuterated water (D_2_O). The ^1^H NMR spectrum of each sample was recorded at 400 MHz with a JEOL-400 spectrometer (JEOL Ltd., Tokyo, Japan).

### 2.5. Statistical Analysis

The bitter taste sensing data were statistically analyzed using the Student’s *t*-test. Differences from the control conditions were considered significant at *p*-values of less than 0.05* and 0.01**.

## 3. Results

**Figure 1:**
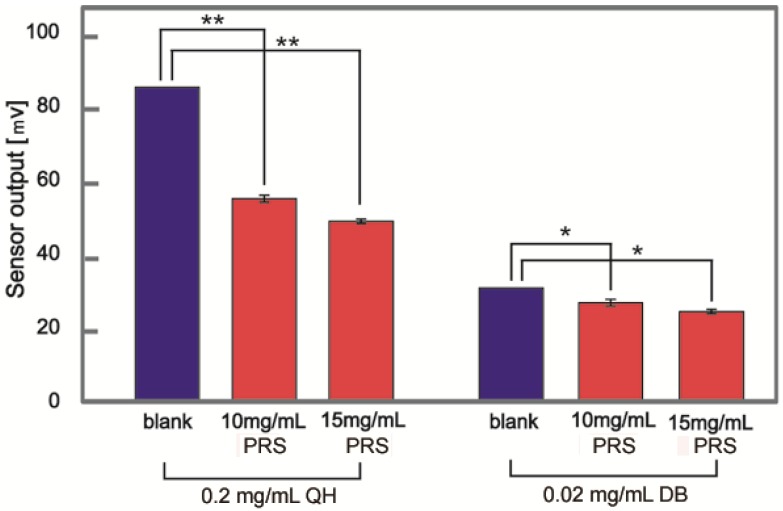
The bitterness of QH and DB in the absence and presence of powdered roasted soybean (PRS) measured as the change in the membrane electric potential of a taste sensor. The reference potential was obtained by immersing the sensor electrode in a 30 mM KCl solution. The bars represent mean ± SD, *n* = 5, * *p* < 0.05, ** *p* < 0.01.

The bitterness of various samples was evaluated using a bitter taste sensor (Figure 1). The sensor output for a 0.2 mg/mL QH solution was 84.9 mV. When 10 and 15 mg/mL of PRS were added to QH, the sensor output for the samples decreased to 54.8 and 48.6 mV, respectively. Similarly, the sensor output of 30.6 mV for a 0.02 mg/mL DB solution decreased to 26.6 and 24.4 mV when 10 and 15 mg/mL PRS, respectively, were added to DB.

**Figure 2:**
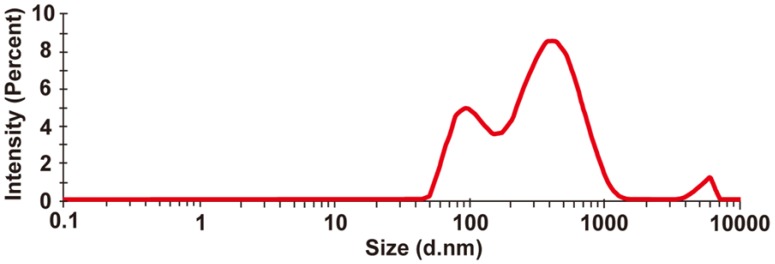
DLS analysis of 1.69 mM PRS extracts in phosphate buffered saline solution (pH 7.4, 0.1 mM).

The extracts of PRS were analyzed using DLS, as shown in Figure 2. The solution of substances extracted from PRS in phosphate buffered saline solution (pH 7.4, 0.1 mM) was found to contain particles of over 100 nm in size.

**Figure 3:**
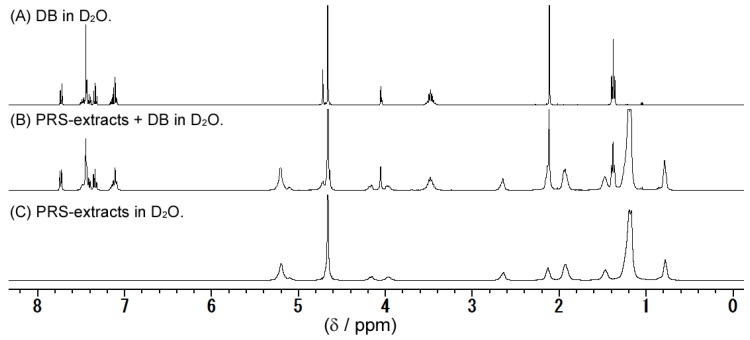
The ^1^H NMR spectra (400 MHz, 298 K, D_2_O). (**A**) DB in D_2_O solution; (**B**) PRS extracts + DB in D_2_O solution; and (**C**) PRS extracts in D_2_O solution.

The ^1^H NMR spectra of DB, the substances extracted from PRS, and a mixture of DB and the extracted substances were measured in aqueous solution, as shown in Figure 3. The DB peaks in the mixture were broadened when compared to those of the DB solution.

## 4. Discussion

Quantitative evaluations of the degree of bitterness using a bitterness sensor showed that PRS significantly suppresses the bitterness of QH and DB. This result indicates that the bitterness taste receptor does not participate in the QH or DB suppression mechanism of PRS. Moreover, the quantitative result was consistent with the previous sensory study [24] on the masking effect of PRS on QH and DB. On the other hand, in the previous sensory study, other powdered foods such as starch did not mask the bitterness, indicating that the bitterness suppression mechanism does not involve physically covering the tongue with powdered solids. Therefore, to further elucidate the bitterness-masking mechanism of PRS, we focused on the ability of PRS to form a suspension in water. The substances in the PRS suspension were extracted using chloroform. The PRS extracts also suppressed the bitterness of QH and DB to a similar extent as that observed for PRS using the bitterness sensor. The PRS extracts were evaluated using DLS (Figure 2), and the suspension was found to consist of particles that were several hundreds of nanometers in size. To evaluate these particles in the suspension of PRS extracts, ^1^H NMR spectra were collected (Figure 3). The observation of several peaks between 0.8 and 2.6 ppm indicated the presence of hydrocarbons derived from fatty acids in PRS extracts. As the suspension is in aqueous solution, these fatty acids are likely derived from phospholipids, which are abundant in PRS. Moreover, the broadening of all the peaks for the PRS extracts indicates that polymerized structures were formed. These NMR and DLS results suggest the formation of liposomes that are several hundreds of nanometers in size by the association of phospholipids [32]. Interestingly, the addition of DB into the suspension of PRS extracts resulted in the broadening of the sharp peaks of DB (1.4, 3.5, 4.1, 4.8, and 7.1–7.8 ppm), similar to the broad PRS extract peaks (Figure 3b). This result indicates that DB interacts with PRS extracts via hydrophobic and ionic interactions [33]. QH is expected to interact with PRS through the same mechanism. Therefore, we think that the bitterness-masking mechanism of PRS might involve the bitterness components becoming entrapped in liposomes formed by the phospholipids of PRS [34,35]. Thus, the bitterness components are physically removed from T2Rs.

In this study, a bitter taste sensor analysis was used to reveal the bitterness-masking effect of PRS. The bitterness-masking mechanism was evaluated by analyzing the masking components extracted from PRS using DLS and NMR. PRS has many components in addition to phospholipids, which may contribute to further masking mechanisms that are not yet understood. However, as the phospholipids of PRS are easily handled and undergo hydrolysis due to lipase present in the duodenum, PRS might be useful as a component of orally administered medicines that are absorbed after leaving the duodenum.

## Abbreviations

The following abbreviations are used in this manuscript:

PRS	Powdered roasted soybeans
T2Rs	Bitter taste receptors
QH	Quinine hydrochloride
DB	Denatonium benzoate
DLS	Dynamic light scattering
NMR	nuclear magnetic resonance
Vr	Reference potential
Vs	Membrane potential

## References

[1] Drewnowski A., Gomez-Carneros C. (2000). Bitter taste, phytonutrients, and the consumer: A review. Am. J. Clin. Nutr..

[2] Chandrashekar J., Hoon M.A., Ryba N.J.P., Zuker C.S. (2006). The receptors and cells for mammalian taste. Nature.

[3] Nakamura T., Tanigake A., Miyanaga Y., Ogawa T., Akiyoshi T., Matsuyama K., Uchida T. (2002). The effect of various substances on the suppression of the bitterness of quinine–human gustatory sensation, binding, and taste sensor studies. Chem. Pharm. Bull..

[4] Squier C.A., Mantz M.J., Wertz P.W. (2010). Effect of menthol on the penetration of tobacco carcinogens and nicotine across porcine oral mucosa *ex vivo*. Nicotine Tob. Res..

[5] Wilkie L.M., Capaldi Phillips E.D., Wadhera D. (2014). Sodium chloride suppresses vegetable bitterness only when plain vegetables are perceived as highly bitter. Chemosens. Percept..

[6] Slack J.P., Brockhoff A., Batram C., Menzel S., Sonnabend C., Born S., Galindo M.M., Kohl S., Thalmann S., Ostopovici-Halip L. (2010). Modulation of bitter taste perception by a small molecule hTAS2R antagonist. Curr. Biol..

[7] Brockhoff A., Behrens M., Roudnitzky N., Appendino G., Avonto C., Meyerhof W. (2011). Receptor agonism and antagonism of dietary bitter compounds. J. Neurosci..

[8] Greene T.A., Alarcon S., Thomas A., Berdougo E., Doranz B.J., Breslin P.A.S., Rucker J.B. (2011). Probenecid inhibits the human bitter taste receptor TAS2R16 and suppresses bitter perception of salicin. PLoS ONE.

[9] Roland W.S.U., Gouka R.J., Gruppen H., Driesse M., van Buren L., Smit G., Vincken J.-P. (2014). 6-Methoxyflavanones as bitter taste receptor blockers for hTAS2R39. PLoS ONE.

[10] Khan S., Kataria P., Nakhat P., Yeole P. (2007). Taste masking of ondansetron hydrochloride by polymer carrier system and formulation of rapid-disintegrating tablets. AAPS PharmSciTech.

[11] Bora D., Borude P., Bhise K. (2008). Taste masking by spray-drying technique. AAPS PharmSciTech.

[12] Yan Y.-D., Woo J.S., Kang J.H., Yong C.S., Choi H.-G. (2010). Preparation and evaluation of taste-masked donepezil hydrochloride orally disintegrating tablets. Biol. Pharm. Bull..

[13] Prajapati S.T., Patel P.B., Patel C.N. (2012). Formulation and evaluation of sublingual tablets containing Sumatriptan succinate. Int. J. Pharm. Invest..

[14] Joshi S., Petereit H.-U. (2013). Film coatings for taste masking and moisture protection. Int. J. Pharm..

[15] Yewale C.P., Rathi M.N., Kore G.G., Jadhav G.V., Wagh M.P. (2013). Formulation and development of taste masked fast-disintegrating tablets (FDTs) of Chlorpheniramine maleate using ion-exchange resins. Pharm. Dev. Technol..

[16] Cantor S.L., Khan M.A., Gupta A. (2015). Development and optimization of taste-masked orally disintegrating tablets (ODTs) of clindamycin hydrochloride. Drug Dev. Ind. Pharm..

[17] Khobragade D.S., Potbhare M.S., Patil A.T. (2016). Evaluation of gum sandarac as a novel release controlling coating polymer for formulation of sustained release pellets. Int. J. Adv. Pharm..

[18] Binello A., Cravotto G., Nano G.M., Spagliardi P. (2004). Synthesis of chitosan–cyclodextrin adducts and evaluation of their bitter-masking properties. Flavour Fragr. J..

[19] Szejtli J., Szente L. (2005). Elimination of bitter, disgusting tastes of drugs and foods by cyclodextrins. Eur. J. Pharm. Biopharm..

[20] Aytac Z., Uyar T. (2016). Antioxidant activity and photostability of α-tocopherol/β-cyclodextrin inclusion complex encapsulated electrospun polycaprolactone nanofibers. Eur. Polym. J..

[21] Katsuragi Y. (1993). Specific inhibitor for bitter taste. Nature.

[22] Tokuyama E., Shibasaki T., Kawabe H., Mukai J., Okada S., Uchida T. (2006). Bitterness suppression of BCAA solutions by L-ornithine. Chem. Pharm. Bull..

[23] United States Department of Agriculture (2016). USDA National Nutrient Database for Standard Reference 28: Soybeans, Mature Seeds, Roasted, no Salt Added.

[24] Makita Y., Ishida T., Kobayashi N., Fujio M., Fujimoto K., Moritomo R., Fujiwara S. (2014). Study on masking effect of kinako on bitterness. Jpn. J. Taste Smell Res..

[25] Akitomi H., Tahara Y., Yasuura M., Kobayashi Y., Ikezaki H., Toko K. (2013). Quantification of tastes of amino acids using taste sensors. Sens. Actuators B.

[26] Tahara Y., Toko K. (2013). Electronic tongues–A review. IEEE Sens. J..

[27] Uchida T. (2014). Comprehensive evaluation of palatability for commercial medicine by taste sensing system. Yakugaku Zasshi.

[28] Preis M., Eckerta C., Häuslerb H., Breitkreutza J. (2014). A comparative study on solubilizing and taste-masking capacities of hydroxypropyl-β-cyclodextrin and maltodextrins with high amylose content. Sens. Actuat. B Chem..

[29] Choi D.H., Kim N.A., Nam T.S., Lee S., Jeong S.H. (2014). Evaluation of taste-masking effects of pharmaceutical sweeteners with an electronic tongue system. Drug Dev. Ind. Pharm..

[30] Haraguchi T., Uchida T., Hazekawa M., Yoshida M., Nakashima M., Sanda H., Hase T., Tomoda Y. (2016). Ability of food/drink to reduce the bitterness intensity of topiramate as determined by taste sensor analysis. Chem. Pharm. Bull..

[31] Wu X., Onitake H., Haraguchi T., Tahara Y., Yatabe R., Yoshida M., Uchida T., Ikezaki H., Toko K. (2016). Quantitative prediction of bitterness masking effect of high-potency sweeteners using taste sensor. Sens. Actuat. B.

[32] Shin K., Maeda H., Fujiwara T., Akutsu H. (1995). Molecular miscibility of phosphatidylcholine and phosphatidylethanolamine in binary mixed bilayers with acidic phospholipids studied by ^2^H- and ^31^P-NMR. Biochim. Biophys. Acta.

[33] Katsuragi Y., Sugiura Y., Lee C., Otsuji K., Kurihara K. (1995). Selective inhibition of bitter taste of various drugs by lipoprotein. Pharm. Res..

[34] Sohi H., Sultana Y., Khar R.K. (2004). Taste masking technologies in oral pharmaceuticals: Recent developments and approaches. Drug Dev. Ind. Pharm..

[35] Tripathi A., Parmar D., Patel U., Patel G., Daslaniya D., Bhimani B. (2011). Taste masking: A novel approach for bitter and obnoxious drugs. J. Pharm. Sci. Biosci. Res..

